# Victims of Fashion

**Published:** 1883-07

**Authors:** 


### VICTIMS OF FASHION.

A SHOE DEALER says that mothers ruin their children's feet through vanity. "One will bring me her baby and ask me to try a pair of shoes on it that will look 'real sweet.' I know what that means, but I'm always sorry for the baby, who is usually in its first short dress, and as skittish as any old maid about having its feet meddled with. I don't say that I'm going to put a shoe on it a size larger than the foot seems to be, but I do; at least I get it on as well as any one could fit a foot operated by a perpetual motion power. Then I trust to the mother's sense for results. If it is her first baby she will be indignant and say that she doesn't want 'the treasure' to 'look sloppy in its shoes.' They must fit exactly or she won't take them. I insist that the child's weight will push the foot out at least a fourth of an inch, and that the shoe is just right. If she objects again I give up and find what she wants. The foot is squeezed into a tight shoe and the baby protests by squalling. She says the seraph is teething or colicky, or hasn't had its usual nap, and she shakes it so vigorously, while declaring the shoes are 'just lovely,' and that its papa will be delighted. The chances are that when she wants another pair she will leave the baby at home and bring down its shoe literally burst out at the toe. She wants several pairs to take home for trial, and I notice that the only ones I considered unsuitable are the very pair she prefers. Children would have better looking feet if they had wiser mothers, and the fault lies in the first shoes worn. One pair too short will ruin the feet, no matter how loose subsequent ones may be. Yes, but after the little people have laid a foundation for corns and bunions. I know many children between two and three years who have both these afflictions because their mothers wanted them to look 'cute,' as they term this phrase of foot squeezing."

